# Controlled Atmosphere Improves the Quality, Antioxidant Activity and Phenolic Content of Yellow Peach during the Shelf Life

**DOI:** 10.3390/antiox11112278

**Published:** 2022-11-17

**Authors:** Xinrui Dong, Yi He, Chushan Yuan, Xiaomei Cheng, Gaoyang Li, Yang Shan, Xiangrong Zhu

**Affiliations:** 1 Longping Branch, College of Biology, Hunan University, Changsha 410125, China; 2 Agricultural Product Processing Institute, Hunan Academy of Agricultural Sciences, Changsha 410125, China; 3 Hunan Provincial Key Laboratory of Fruits and Vegetables Storage, Processing and Quality Safety, Changsha 410125, China; 4 Hunan Province International Joint Laboratory on Fruits and Vegetables Processing, Quality and Safety, Changsha 410125, China

**Keywords:** controlled atmosphere, yellow peach, shelf life, antioxidant activity, phenolic content

## Abstract

Controlled atmosphere (CA) has been demonstrated to maintain the shelf-life quality of fruits, but its effect on the antioxidant activities and phenolic content of yellow peach is not comprehensive. This study analyzed the role of CA on the quality of shelf period, phenolic content and antioxidant activity of “Jinxiu” yellow peach. Yellow peach was left under specific aeration conditions (3.5–4% CO_2_, 2–3% O_2_, 92–95.5% N_2_, 1 ± 0.5 °C) and the control (1 ± 0.5 °C) for 21 d, to observe changes in physiological parameters of the fruit during 10 d of the shelf life (25 ± 1 °C). The result showed that CA reduced the weight loss rate (WLR), decay rate (DR), and browning index (BI) of yellow peaches. Furthermore, the CA held a high level of total flavonoid content (TFC), total phenol content (TPC) and phenolic content in the fruit. Antioxidant analysis showed that polyphenol oxidase (PPO) enzyme activity was lower and free radical scavenging capacity (DPPH, ABTS, and FRAP) and antioxidant enzyme activities (POD and PAL) were higher in the CA group. Combining the results of significance analysis, correlation analysis, principal component analysis (PCA) and hierarchical cluster analysis (HCA) clearly identified the differences between the CA group and the control group. The results showed that the CA could maintain higher phenolic content and reduce the oxidation of yellow peach fruit and enhance fruit quality by affecting the antioxidant activities of yellow peach.

## 1. Introduction

Peach (*Prunus persica Batsch.*) is a drupe-like fruit that grows mainly in temperate and subtropical regions [1]. China is one of the major peach producers in the world. There are three types of peaches: red, white, and yellow peach [2]. Among them, yellow peaches have received more attention from consumers because of their unique organoleptic properties and high nutritional value [3]. However, the high metabolism and high decay rate limit the market circulation of yellow peach. Cold storage is the most frequent method to delay the deterioration of peach fruit, while long-term cold storage (below 8 °C) leads to chilling injuries (CI) such as internal browning, flesh leatheriness, and loss of sensory-related compounds [4,5]. Therefore, it is imperative to choose a suitable postharvest preservation method to maintain the quality of yellow peaches and inhibit the occurrence of CI.

Controlled atmosphere (CA) [6,7] has shown the potential to reduce the cold sensitivity of most tropical fruits [8,9,10] and extend the shelf life of many horticultural crops [11,12]. Jeremy et al. reported that CA conditions of 2% O_2_, 2% CO_2_, and 5 °C maintained fruit appearance and retarded mid-fruit decay in avocados [13]. Similarly, E Paulsen et al. showed that modified atmosphere packaging (MAP) storage extended the shelf life and improved the sensory quality of cherry tomatoes [14]. These beneficial effects obtained by CA were achieved by reducing oxygen levels and inhibiting metabolism [15]. SE Youssef et al. showed that CA with 2% O_2_ and 15% CO_2_ controlled reduced the incidence and severity of peach decay [16]. However, the mechanisms driving the extension of the shelf life of peaches and improving the shelf-life quality have not been clearly elucidated [17].

Phenols are important secondary metabolites of peach fruit that play a role in reactive oxygen species detoxification, disease response, and defense effects [18]. The antioxidant and ROS scavenging abilities of bioactive substances confer disease resistance to peach fruit [19]. However, these compounds are gradually lost during postharvest storage by oxygen and polyphenol oxidase. CA significantly reduced the loss of phenolic compounds and antioxidant capacity in lychee [20], apple [21], and goji berry [22]. However, few studies have been conducted on the effect of CA-treated peach fruit on phenolic substances during the shelf life.

This study investigated the bioactive substance content, antioxidant capacity, and some important quality indicators such as weight loss rate (WLR), decay rate (DR), browning index (BI), firmness and color difference of CA-treated yellow peach on 0, 2, 4, 6, 8, and 10 d of the shelf life. Finally, this work used significance analysis, correlation analysis, principal component analysis (PCA), and hierarchical cluster analysis (HCA) to fully understand the changes in bioactive substances, antioxidant capacity, and postharvest quality of yellow peach during the shelf life and their interrelationships.

## 2. Materials and Methods

### 2.1. Plant Material and Controlled Atmosphere (CA) Treatment

Yellow peach (“Jinxiu”) was purchased from a fruit store in Changsha, Hunan Province, in August 2021 and immediately transferred to the cold storage of the Hunan Academy of Agricultural Sciences. Fruits with defects such as mechanical damage, black spots, pests, and diseases were excluded, and 300 selected yellow peach fruits were divided into two groups and placed in air and 3.5–4% CO_2_, 2–3% O_2_, and 92–95.5% N_2_ atmospheres, respectively. Samples were placed at 1 ± 0.5 °C for 21 d and then transferred to a shelf environment (25 ± 1 °C), where WLR, DR, BI, firmness, and color difference were investigated every other day during the shelf life. The fruits were also rapidly frozen with liquid nitrogen and stored at 80 °C for the rest of the index analysis. In the experiment, three replicates were analyzed.

### 2.2. Assay of Weight Loss Rate (WLR)

WLR of the fruit is measured by the following equation

(1) $$\mathrm{Weight}\;\mathrm{loss}\;\mathrm{rate}\;\left(\%\right)=\frac{\mathrm{IFW}-\mathrm{FFW}}{\mathrm{FFW}}\times 100$$

The initial fruit weight is represented by IFW, and the final fruit weight is represented by FFW. WLR was assessed using a total of 10 fruits per replicate.

### 2.3. Assay of Browning Index (BI)

Based on the brown area on the surface of the fruit, the fruit was tested for four levels of decay, namely [23], Class 0, browning area = 0%; Class 1, 1% < browning area ≤ 25%; Class 2, 26% < browning area ≤ 50%; Class 3, 51% < browning area ≤ 75%; Class 4, 76% < browning area ≤ 1. The BI is obtained as follows:

(2) $$\mathrm{Browning}\;\mathrm{index}\;\left(\%\right)=\frac{\Sigma \left(\mathrm{Browning}\;\mathrm{grade}\;\times \mathrm{number}\;\mathrm{of}\;\mathrm{fruit}\right)}{4\times \mathrm{total}\;\mathrm{number}\;\mathrm{of}\;\mathrm{fruit}\;\mathrm{in}\;\mathrm{treatment}}\;$$

### 2.4. Assay of Firmness

Six neatly shaped (spherical) yellow peaches of uniform size (250 ± 15 g) from both the control and the CA group were used for the determination of firmness. The sample firmness was obtained by the CT3 food texture analyzer with a 5 mm diameter cylindrical probe at a rate of 1 mm·s^−1^. All experiments were performed with 1 cm from the equatorial line of each sample, the maximum force during penetration is read and the unit was expressed as grams (g).

### 2.5. Assay of the Decay Rate (DR)

Fruits were assessed for visible decay and the number of fruits with a decayed surface area exceeding 1 cm^2^ was recorded as the number of bad samples. The DR is measured by equal percentage.

(3) $$\;\mathrm{The}\;\mathrm{decay}\;\mathrm{rate}\;\left(\%\right)=\frac{\mathrm{Number}\;\mathrm{of}\;\mathrm{bad}\;\mathrm{fruits}}{\mathrm{Total}\;\mathrm{number}\;\mathrm{of}\;\mathrm{fruits}}\;$$

### 2.6. Assay of Color

The color attributes were tested at three points of the equatorial position by the colorimeter and were expressed as brightness L *, a *, and b *. The measurement is repeated three times for each color parameter and results were expressed as mean value.

The total chromatic difference (∆E) is calculated using Formula (4). Based on the different degrees of yellow peach color, the ∆E is divided into three levels [24], 0.0 < ∆E ≤ 2.0 corresponds to Class 0, representing color difference is almost invisible to human vision; 2.0 < ∆E ≤ 3.5 corresponds to Class 1, which means that most people cannot identify color difference and professionally trained people can identify it; 3.5 < ∆E corresponds to Class 2, representing color difference can be distinguished. The ∆E is obtained as follows:

(4) $$\;\mathrm{The}△E=\sqrt{{\left(L_{1}{}^{*}-L_{0}{}^{*}\right)}^{2}+{\left(a_{1}{}^{*}-a_{0}{}^{*}\right)}^{2}+{\left(b_{1}{}^{*}-b_{0}{}^{*}\right)}^{2}}$$

In the formula, where L_0_*, a_0_*, and b_0_* are the color coordinates of the reference sample (the yellow peach before treatment), and L_1_*, a_1_*, and b_1_* are the color coordinates of the yellow peach during the shelf life. 

### 2.7. Assay of DPPH, ABTS and FRAP Scavenging Activity

The DPPH (2,2-diphenyl-1-picrylhydrazyl) was recorded by spectrophotometer based on previous method [25]. Yellow peach samples (5.0 g) were ground with 25 mL 80% (*v*·*v*^−1^) ethanol and centrifuged for 30 min (4 °C, 16,000 rpm), then the upper clear liquid was extracted for analysis. The mixture was treated in the dark at 26 °C for 35 min and the photometric value at 517 nm was recorded, The DPPH was measured by the equation.

(5) $$\mathrm{DPPH}\;\mathrm{radical}\;\mathrm{scavenging}\;\mathrm{activity}\;\left(\%\right)=\frac{\left(A_{0}-A_{1}\right)}{A_{0}}\times 100$$

The ABTS (2,2′-azino-bis(3-ethylbenzthiazoline-6-sulphoic acid)) test was performed with reference to the method of VEGARA S et al. [26]. The working fluid was provided by pouring 2.45 mmol L^−1^ potassium persulfate and 7 mmol L^−1^ stock solution containing 20 mmol·L^−1^ acetate buffer (pH 4.5) in equal quantity and incubated at 37 °C for 18 h. The 3.6 mL of ABTS working solution and 0.4 mL of sample extraction solution or standard was mixed in an assay tube and reacted for 30 min protected from light and measured at 510 nm. The ABTS radical scavenging rate was obtained as the formula:

(6) $$\mathrm{ABTS}\;\mathrm{radical}\;\mathrm{scavenging}\;\mathrm{activity}\;\left(\%\right)=\frac{\left(A_{0}-A_{1}\right)}{A_{0}}\times 100$$

The FRAP (ferric ion reducing antioxidant power) assay was measured according to the method established in the literature [27]. The TPTZ working solution was obtained by adding 25 mL acetate buffer (pH 3.6), 2.5 mL 10 mmol·L^−1^ TPTZ solution, and 2.5 mL 20 mmol·L^−1^ FeCl_3_. An amount of 100 μL supernatant was added to 4 mL of the TPTZ working solution, The mixed fluid was maintained at 37 °C for 10 min and the optical density value at 593 (OD_593_) was determined.

(7) $$\mathrm{FRAP}\;\mathrm{radical}\;\mathrm{scavenging}\;\mathrm{activity}\;\left(\%\right)=\frac{\left(A_{0}-A_{1}\right)}{A_{0}}\times 100$$

In Equations (5)–(7), where *A*_0_ is the absorbance value of the fruit sample, and *A*_1_ is the absorbance value of the blank fruit.

### 2.8. Assay of Phenylalanine-Catalase Activity (PAL)

The PAL activity was analyzed through the approach by MAROGA et al. [28]. The enzyme reaction system consisted of 0.1 mL of enzyme substance, 16 mmol·L^−1^ phenylalanine, 3.6 mmol L^−1^ NaCl, 50 mmol·L^−1^ Tris-HCI (pH 8.8), and 980 μL of 6 mol·L^−1^ hydrochloric acid. The 6 mmol L^−1^ hydrochloric acid was used as a control tube. Each gram of sample tissue in each milliliter of the reaction system causes a change in the photometric value at 290 nm of 0.1 per minute as one unit of enzyme activity.

### 2.9. Assay of Polyphenol Oxidase Activity (PPO)

The PPO activity was measured with the method of Shi et al. [29]. The enzyme was extracted from 4% polyvinylpyrrolidone (PVPP) and acetate-sodium acetate buffer (pH 5.9). The enzyme reaction system contained enzyme extract, 0.05 mmol·L^−1^ (pH 5.9) phosphate buffer, and 0.1 mol·L^−1^ catechol solution. Boiled sample and 20% trichloroacetic acid were used as controls. The difference of 0.01 per minute between the OD_525_ values of the two tubes was considered as one unit of enzyme activity.

### 2.10. Assay of Peroxidase Activity (POD)

The activity of POD was measured as used by Li et al. [30]. The enzyme reaction system consisted of 20 mmol·L^−1^ KH_2_PO_4_, 100 mmol·L^−1^ phosphate buffer solution (pH 6.0), guaiacol, and 19 μL 30% hydrogen peroxide, and then the absorbance value of the reaction mixture was measured at 470 nm, and the amount of enzyme required to cause a 0.01 change in the 0D_470_ value in one minute was defined as one unit of POD activity.

### 2.11. Assay of Total Flavonoid Content (TFC)

The TFC estimates refer to the method used by XIE Y et al. [31]. The samples were extracted with 80% ethanol. The OD_510_ was measured, and the results were expressed as mg Rutin equivalent per gram of fresh weight.

### 2.12. Assay of Total Phenolic Content (TPC)

The assay of TPC was carried out using the method of RADUNIC M et al. [32]. Total phenolic was extracted using 80% ethanol solution, the OD_765_ was measured after 60 min of reaction at room temperature. The TPC was expressed as gallic acid equivalents (mg·g^−1^ FW) per gram of fresh fruit weight.

### 2.13. Assay of Phenolic Acids Content

The determination of phenolic acids was performed with some modifications based on Tounsi et al. [33]. Phenolic acids in the samples were extracted with 1% ethanolic acid solution and filtered through a microporous membrane (0.22 μm). The characterization and quantification of phenolic acids were performed on a Shimadzu HPLC system (LC-10AT) using a C18 column (4.6 mm × 250 mm, 5 µm) at a rate of 1.0 mL min^−1^, and the column temperature was set at 40 °C. The mobile phases A and B were chromatographic grade acetonitrile and chromatographic grade 4% acetic acid. Sample (10 μL) was eluted in a gradient for 39 min, 0–28 min, 0–10% A; 28–29 min, 10–45% A; 29–39 min, 45–90% A. The detection wavelength of PDA was set at 280 nm and 520 nm. The concentration of phenolic acids was expressed in µg·g^−1^.

### 2.14. Statistical Analysis

All data reflected as mean ± standard error (SE) of three bio-repeats. The data obtained from the experiment were parsed by PCA and HCA. ANOVA was performed using Statistical Product Service Solutions (SPSS) (version 26) from IBM (Chicago), *p*-value not greater than 0.05 were regarded as a significant difference.

## 3. Results

### 3.1. Color

**Figure 1:**
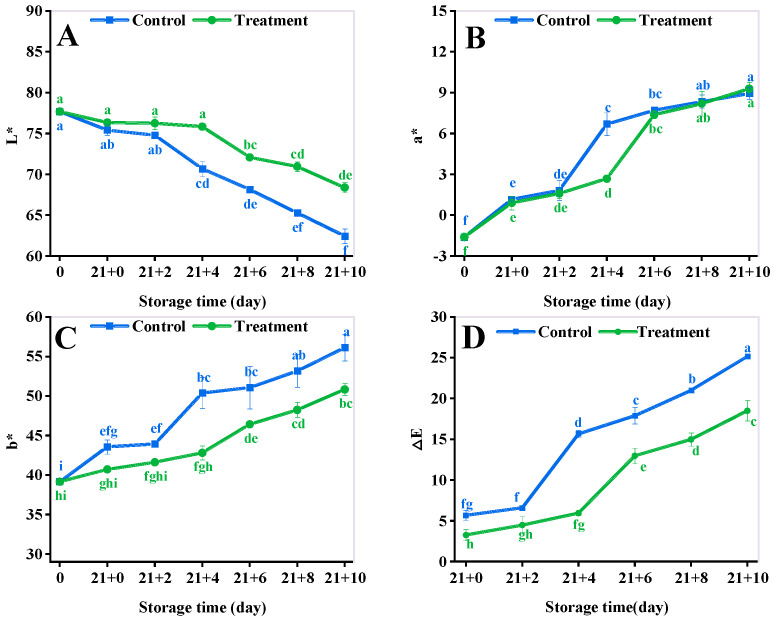
Effect of different storage methods on the L* (**A**), a* (**B**), b* (**C**), and ∆E* (**D**) of yellow peaches. Vertical bars represent standard deviations. Different letters represent statistical differences (*p* < 0.05).

Color is one of the important indicators to characterize the change in the freshness of fresh fruits. The sensory quality attributes of yellow peach continued to decrease during the shelf life. The changing trend of color parameters of CA and the control group was similar, mainly showing a decrease in L* values (Figure 1A) and a continuous increase in a* values (Figure 1B), b* values (Figure 1C), and ∆E (Figure 1D). The L* values of the CA group increased by 9.5% (*p* < 0.05) compared with that of the control at 10 d, and a* and b* of the treatment group increased by 150% and 17.68% (*p* < 0.05), respectively, compared to that of the control at 4 d, and the ∆E of the treatment group decreased by 26.45% (*p* < 0.05) compared with that of the control group at 10 d which indicated that CA significantly delayed the color change in yellow peach.

### 3.2. Browning Index (BI)

**Figure 2:**
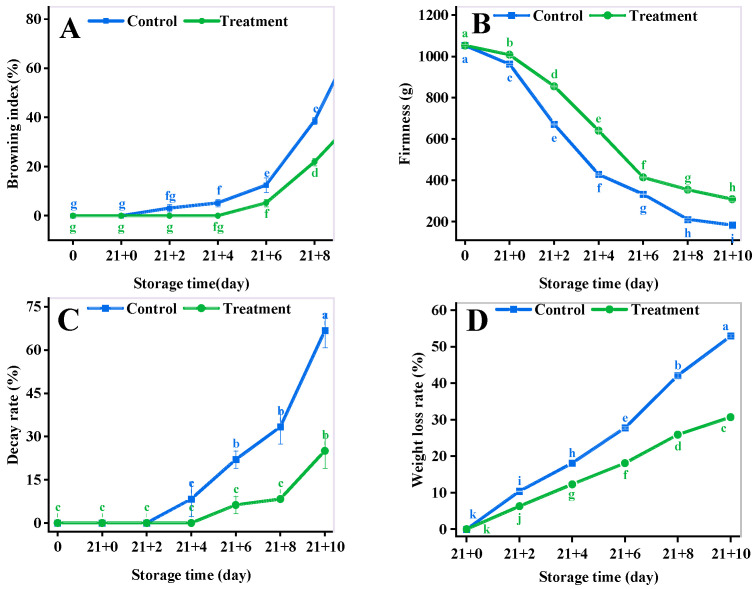
Effect of different storage methods on browning index (**A**), firmness (**B**), decay rate (**C**), and weight loss rate (**D**) of yellow peaches. Vertical bars represent standard deviations. Different letters represent statistical differences (*p* < 0.05).

The BI gradually increased with the extension of the shelf life of yellow peach (Figure 2A). The BI of the treatment fruit varied slightly from 0 d to 2 d, and then rapidly rise from 2 d to 4 d. The BI of the control and the treatment were 12.5% and 5.21%, respectively, at 6 d (*p* < 0.05). The BI of the CA treatment was 62.5% less than that of the control at 10 d. The results showed that CA significantly delayed fruit browning during the shelf life.

### 3.3. Firmness

Firmness is one of the representative indicators reflecting the quality of the fruit [34]. The firmness of the control and the CA treatment was significantly lower than the initial value of 59.24% (*p* < 0.05) and 18.77% (*p* < 0.05) at 4 d, respectively (Figure 2B). The firmness of the CA treatment was 69% higher than that of the control at 10 d of shelf life. It indicated that the treatment group significantly reduced the decrease in the firmness of yellow peach.

### 3.4. The Decay Rate (DR)

The DR of fruit in the treatment group was significantly lower than that of the control (*p* < 0.05) (Figure 2C). The DR of the control and the CA treatment was 22.92% and 6.25% (*p* < 0.05), respectively, at 6 d, and the CA treatment decreased by 72.73% compared to that of the control (*p* < 0.05) at 6 d. The difference between the two groups was greatest at 10 d, with a 60% decrease in the DR in the CA treatment compared to that of the control. 

### 3.5. The Weight Loss Rate (WLR)

The WLR of both groups increased rapidly during the shelf life (Figure 2D). The WLR of the control and the CA treatment was 1.04% and 0.63%, respectively, at 2 d, and the two groups were statistically significant (*p* < 0.05). The WLR of the CA treatment showed a 41.94% reduction compared to that of the control at 10 d (*p* < 0.05). 

### 3.6. Browning-Related Enzymes

**Figure 3:**
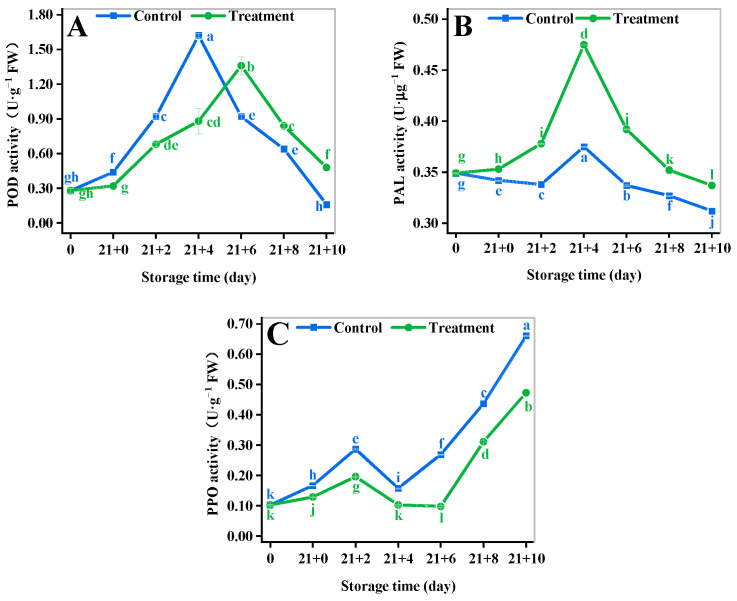
Effect of different storage methods on POD (**A**), PAL (**B**), and PPO (**C**) enzyme activities of yellow peach. Vertical bars represent standard deviations. Different letters represent statistical differences (*p* < 0.05).

The POD activity of the fruit showed an increasing and then decreasing trend (Figure 3A). The POD activity of the CA treatment and the control reached a peak at 4 d and 6 d, respectively. The POD activity of the CA treatment increased by 200% to that of the control at 10 d. It indicated that the treatment group maintained a stronger level of POD activity during the shelf life.

The PAL activity in both two groups showed a slight increase at the beginning and peaked at 4 d (Figure 3B). The PAL activity of CA treatment increased by 26.5% compared to that of the control at 4 d (*p* < 0.05). The PAL activity of CA treatment increased by 8.01% than that of the control at 10 d (*p* < 0.05). The PAL activity of the treatment group was higher than that of the control group throughout the shelf-life period.

The trends of PPO activity in the two groups during the shelf life were the same and the PPO activity of the CA treatment was always statistically significantly lower than that of the control (Figure 3C). The minimum value of PPO in the CA treatment was reduced by 35.09% compared to that of the control (*p* < 0.05). The PPO of CA treatment was reduced by 28.28% compared to that of the control at 10 d of the shelf life (*p* < 0.05). It indicated that CA plays an important role in reducing the incidence of browning by inhibiting PPO activity.

### 3.7. Total Phenolic Content (TPC)

**Figure 4:**
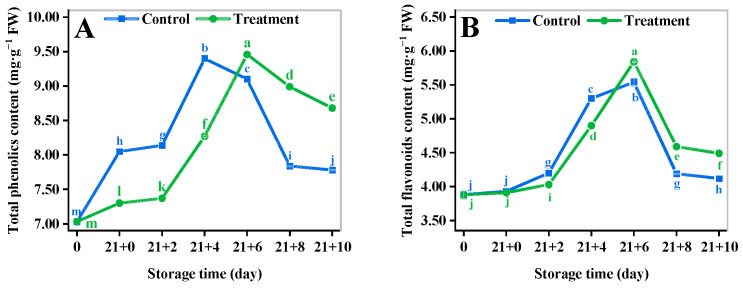
Effect of different storage methods on TPC (**A**) and TFC (**B**) of yellow peach. Vertical bars represent standard deviations. Different letters represent statistical differences (*p* < 0.05).

Phenol is a key indicator of fruits and vegetables, and it provides high antioxidant potential [35]. The TPC of the control and the CA treatment reached the peak at 4 d and 6 d, respectively, and the TPC of the control decreased faster than that of the CA-treated in the late shelf life (Figure 4A). The TPC of the CA treatment was 11.58% higher than that of the control at 10 d, and the difference was statistically significant (*p* < 0.05). 

### 3.8. Total Flavonoid Content (TFC)

The trend of the TFC change is similar to that of the TPC, which presented a fluctuation of increasing and then decreasing (Figure 4B). The TFC in CA treatment first increased and reached the maximum level at 6 d, when the TFC of the treatment group was significantly higher than that of the control, and then began to decline, and the TFC in the CA treatment was 8.98% higher than that of the control at 10 d. The results showed that the CA could significantly inhibit the decrease in the TFC. 

### 3.9. Phenolic Acid Content

**Figure 5:**
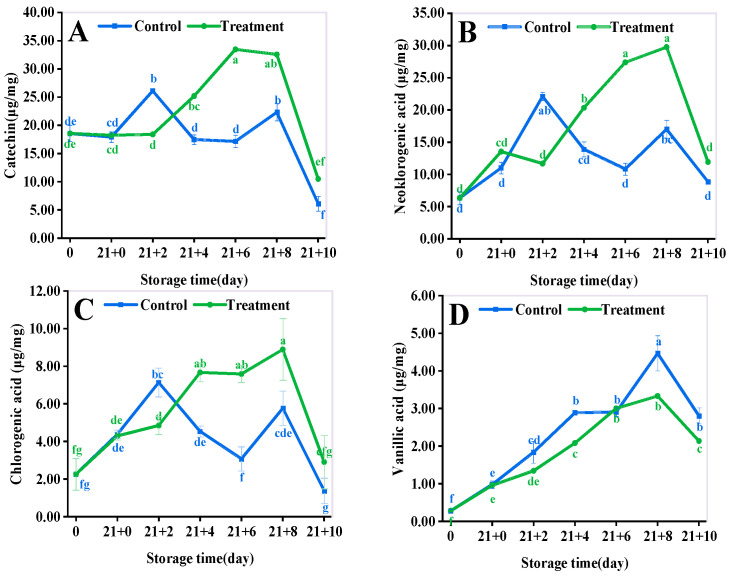
Different storage methods on yellow peach catechin (**A**), neochlorogenic acid (**B**), chlorogenic acid (**C**), and vanillic acid (**D**). Vertical bars represent standard deviations. Different letters represent statistical differences (*p* < 0.05).

**Figure 6:**
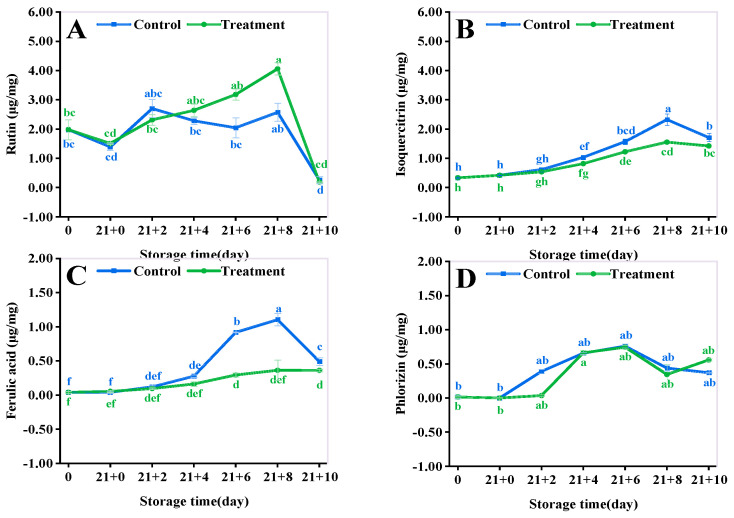
Different storage methods on yellow peach rutin (**A**), isoquercitrin (**B**), ferulic acid (**C**), and phlorizin (**D**). Vertical bars represent standard deviations. Different letters represent statistical differences (*p* < 0.05).

The changes in eight phenolic acids including catechin (Figure 5A), neochlorogenic acid (Figure 5B), chlorogenic acid (Figure 5C), vanillic acid (Figure 5D), rutin (Figure 6A), isoquercitrin (Figure 6B), ferulic acid (Figure 6C) and phlorizin (Figure 6D) in yellow peaches were analyzed during the shelf life. The total phenolic acids were significantly increased in the treated group compared to those of the control group (*p* < 0.05). The phenolic acids in yellow peach were mainly neochlorogenic acid, catechin, and chlorogenic acid, which accounted for about 84.48% of the total phenolic acids. The content of catechin, neochlorogenic acid, and chlorogenic acid increased by 71.95%, 110.59%, and 71.95% compared with that of the CA treatment at 10 d (*p* < 0.05). While vanillic acid, rutin, and isoquercitrin of the control group were less abundant and did not change significantly compared with those of the CA treatment (*p* > 0.05). In addition, the content of ferulic acid in the CA treatment was lower than that in the control. The content of root bark glycosides was higher than that in the control in the early stage, while it was not much different from that in the middle and late stages. The results showed that the phenolic acid content in the CA treatment was consistently higher than that in the control during the shelf life.

### 3.10. Antioxidant Activity Assay

**Figure 7:**
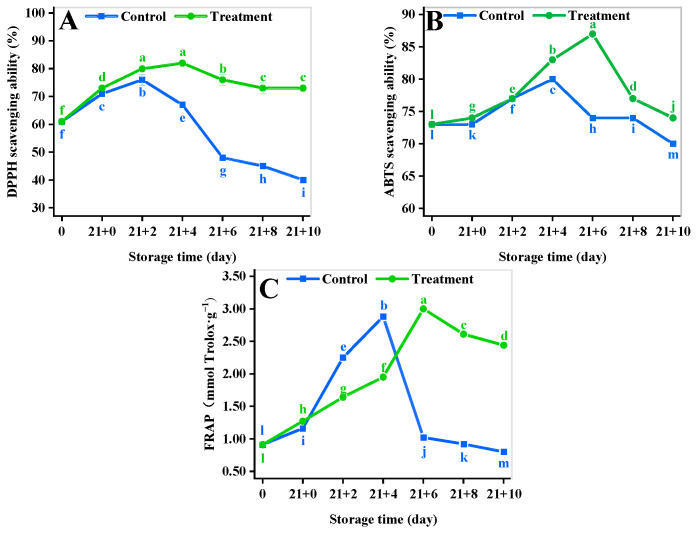
Effect of different storage methods on DPPH (**A**), ABTS (**B**), and FRAP (**C**) of yellow peach. Vertical bars represent standard deviations. Different letters represent statistical differences (*p* < 0.05).

The DPPH, ABTS, and FRAP radical scavenging rate of fruits and vegetables is mainly used for antioxidant evaluation [36] and reflects the trend of their resistance to adversity stress. As shown in Figure 7A, the DPPH free radical scavenging rate of the treatment group was always significantly higher than that of the control (*p* < 0.05). The DPPH radical scavenging rate of the CA treatment increased by 80.86% compared with that of the control (*p* < 0.05) at 10 d of the shelf life. 

The ABTS showed an increasing trend during the shelf life (Figure 7B), with the control reaching a maximum value of 80.12% at 4 d, which significantly decreased to 70.34% at 10 d (*p* < 0.05). The CA treatment delayed the peak by 86.79% until 6 d. The ABTS decreased to 73.54% at 10 d of the shelf life, which was 4.55% greater than that of the control (*p* < 0.05).

The FRAP radical scavenging capacity of both groups showed a rise followed by a decline (Figure 7C). The FRAP maximum of the CA treatment increased by 15.38% compared to that of the control at 4 d. The FRAP value in the CA treatment increased by 203.82% compared to that of the control at 10 d of the shelf life (*p* < 0.05).

### 3.11. Correlation Analysis

**Figure 8:**
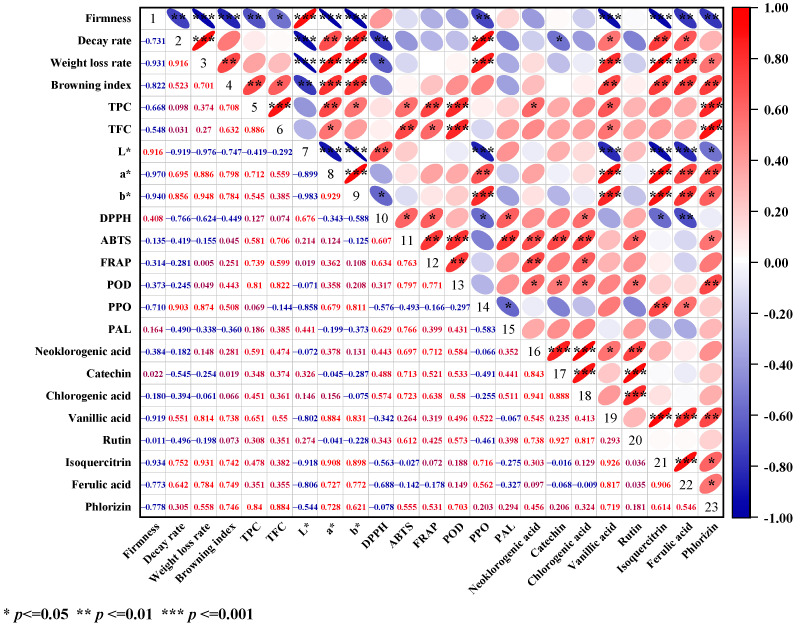
Heat map of correlation analysis of yellow peach index parameters for different treatments, with three replicates for each treatment.

The Pearson correlation method [37] was used to investigate the relationship between the quality indicators of yellow peach treated with CA during the shelf life (Figure 8). The WLR showed a significant positive correlation with PPO enzyme (r^2^ = 0.874) and BI (r^2^ = 0.700) and a highly significant negative correlation with fruit firmness (r^2^ = −0.931). This suggested that PPO enzymes were influenced by water loss rate (WLR) in fruit [38]. It is supported that water stress caused by water loss led to disruption of cell membrane structure [39]; damage to cell membrane structure is the direct cause of fruit browning [40]. The negative correlation between fruit weight loss and PAL and POD enzymes was not significant, indicating that PAL and POD enzymes are influential factors rather than determinants of fruit browning [41]. The WLR was positively correlated with a * (r^2^ = 0.886) and b * (r^2^ = 0.948) and significantly negatively correlated with L *(r^2^ = −0.976). This supported our previous discussion that increased WLR accelerated the flesh browning and eventually led to the darkening of fruit brightness and the increase in DR [42]. 

In addition, the DR was negatively correlated with the content of asam vanilat (r^2^ = 0.551), isoquercitrin (r^2^ = 0.752), and asam ferulat (r^2^ = 0.642), indicating that fruit decay was accompanied by the loss of phenolic substances [43]. Total phenol was significantly and positively correlated with ABTS (r^2^ = 0.580) and FRAP (r^2^ = 0.739) and total flavonoid with ABTS (r^2^ = 0.706) and FRAP (r^2^ = 0.599), but all were insignificantly and positively correlated with DPPH, indicating not only the weak DPPH radical scavenging ability of total phenol and total flavonoid [44], but conferring the fruit stronger ABTS and FRAP value. It also supported our previous discussion that the phenolic content was closely related to the antioxidant capacity of fruits [45]. 

### 3.12. Principal Component Analysis (PCA)

**Figure 9:**
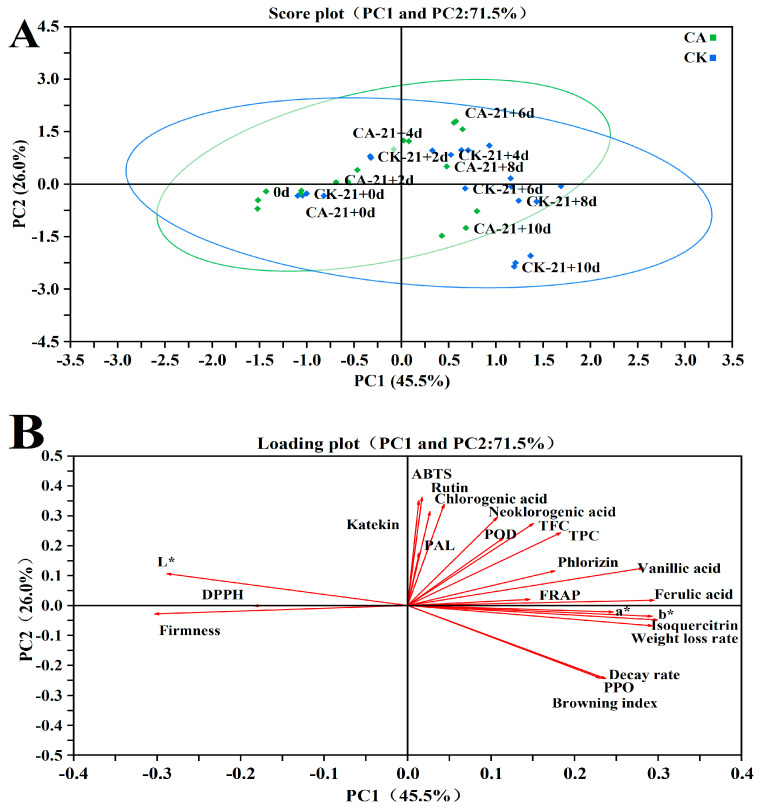
Principal component analysis score plot (**A**) and loading plot (**B**) of yellow peach index parameters for different treatments with three replicates for each treatment.

In order to better compare the parameters for assessing the differences and quality of the shelf life before and after CA, PCA was performed using 23 factors, and the cumulative variance contribution of the first two principal components reached 71.5% (PC1: 45.5% and PC2: 26.0%) (Figure 9A). The following information could be derived from the distribution of 95% confidence ellipses in coordinates for both samples (Figure 9B): (i) the sample points were more clustered within the CA treatment compared to the control, indicating that the CA treatment maintained better quality during the shelf life. (ii) The sample points were independently clustered and had a certain distance between parallel, indicating that the control and the CA treatment could be distinguished from each other at the same time point. Based on the principle of using the eigenvalue >1.0000 as the principal component factor, we can further observe that a total of five principal components were obtained after the dimension reduction of 23 quality indicators by principal component analysis, and the eigenvalues are 10.3074, 5.7969, 2.5247, 1.3025, and 1.0089, respectively. The cumulative variance contribution rate was 91.04%; it indicates that these five principal components cover 91.04% of the total amount of raw data information. As a result, the five main components can be used as comprehensive variables to evaluate the influence of CA on the quality of yellow peach during their shelf life. Combined with the loading plot (Figure 9B), WLR, a* and b* are the main influencing factors of PC1, ABTS, katekin, rutin, asam klorogenat, asam neoklorogenik are the main influencing factors of PC2, the former mainly reflects the maturity of the fruit, while the latter is clearance capacity of free radicals and phenolic acids that mainly reflect the antioxidant capacity of the fruit. The first two principal components cover the physiological and biochemical indicators of the yellow peach samples, so the principal component evaluation of the experiment is scientific and reliable. Furthermore, the variance contribution of PC1 was higher than that of PC2 (45.5% PC1 and 26.0% PC2), so the first principal component characterizing the maturity of fruit during shelf-life quality was more important than the second principal component characterizing fruit antioxidant capacity. 

### 3.13. Hierarchical Clustering Analysis (HCA)

**Figure 10:**
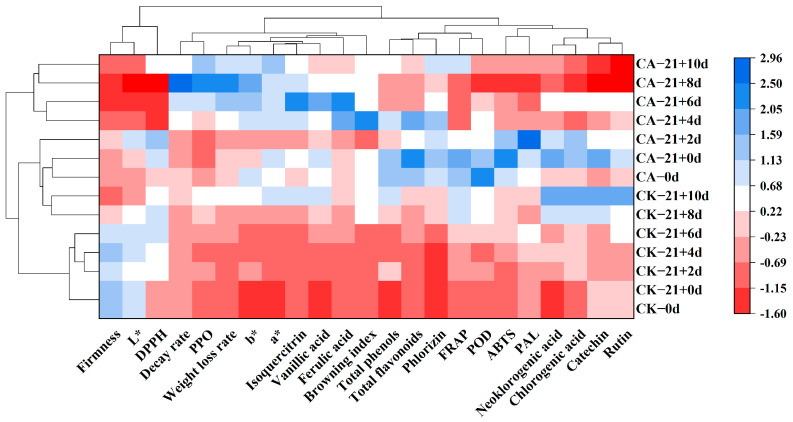
Heat map of hierarchical cluster analysis of yellow peach index parameters for different treatments, with three replicates for each treatment.

The samples and 23 quality indexes were grouped using HCA in order to visualize the degree of good and bad quality of yellow peach during the shelf life and the trend of the change. From Figure 10, samples 0 d, CK−21 + 0 d, CA−21 + 0 d, and CK−21 + 2 d were clustered into group 1, samples CK−21 + 2 d, CK−21 + 4 d, CA−21 + 6 d and CA−21 + 8 d were clustered into group 2, and CK−21 + 6 d, CK−21 + 8 d, CK−21 + 10 d and CA−21 + 10 d were clustered into group 3. The distribution of color bands showed that there was a significant difference in the quality of the samples before and after CA in the middle and late shelf life, and the quality of the two groups showed the same trend with the extension of the shelf life. For the quality indicators, fruit firmness, L*, and DPPH were clustered into group 1, and the remaining 20 indicators related to fruit nutritional quality and antioxidants were clustered into group 2, which were basically consistent with the PCA results. 

## 4. Discussion

Commercial procedures regulate peach storage technology protocols by improving postharvest storage techniques for yellow peach. In addition, there is currently a growing interest in replacing traditional handling methods with simple, green, and efficient methods [46]. CA is a storage method to preserve freshness by regulating the air component in the environment [47]. It can keep fruits and vegetables in a semi-dormant state, which helps to slow down the aging and deterioration process and achieve the purpose of preserving freshness and corrosion [48]. Previous results in fruits such as mangoes [49] and pears [50] showed that CA delayed the loss of fruit during the shelf life and attributed this to the positive effect of CA on fruit stabilization and maintenance of cellular integrity [51]. Similar results were found in our study, where CA significantly reduced the rate of decay of yellow peach during the shelf life.

Color is a visual indicator of fruit ripeness and reflects the shelf-life quality of fresh fruit [52]. During this experiment, it was observed that CA treatment significantly inhibited the glossy darkening and yellowing of yellow peach fruit, mainly reflected in the ∆E of the control group were higher compared to that of the control group during the shelf life, because the CA treatment reduced the oxygen concentration in the storage environment, which inhibited the color change in the yellow peach. This result is also supported by other studies, for example, James et al. found that CA increased the L* value of pears [53]. Nakano K et al. reported that low O_2_ in retarding color development was more effective [54]. This is due to the fact that high CO_2_ inhibited the amount of carotenoid synthesis during fruit ripening [55]. Overall, CA helps to retain the L* values of yellow peach and improves the sudden increase in a*, and b* values during the shelf life.

Water is an indispensable substrate for organisms to carry out metabolic activities [56] and maintain the freshness of fruits and vegetables after harvest [57]. LI et al. reported that fruit softening is closely related to cellular water loss and CA prevented the softening by inhibiting fruit respiration in apples and pears [58,59,60]. Our results also confirmed that short-term CA treatments are effective in reducing the WLR [61] and firmness [62] of fresh yellow peach during the shelf life.

The enzymatic antioxidant system is the main way to control oxidative senescence in fruit. PPO, POD, and PAL are key enzymes that regulated the oxidative browning of fruit [63]. In the present study, CA was able to maintain low PPO activity and high PAL and POD activity in yellow peach for a longer period of time, effectively maintaining the dynamic balance of the reactive oxygen species [64]. On the other hand, the levels of DPPH, ABTS, and FRAP of yellow peach were increased by CA treatment, which further indicated that CA helped to alleviate oxidative browning of yellow peach. Therefore, CA can improve the internal defense capacity and antioxidant level [65] of yellow peach tissue by regulating oxidative browning [66].

In addition, the antioxidant capacity is also related to the accumulation of phenolic compounds, which can evaluate the maturity and determine the antioxidant capacity of fresh fruits and vegetables [67,68]. The main phenolic acids of yellow peach are neochlorogenic acid, catechins, and chlorogenic acid, which bear the non-enzymatic antioxidant activity fraction of the fruit as endogenous antioxidants [67,69], and those are responsible for tolerance to biotic and abiotic stresses [70]. In the present study, the content of TFC and TPC of yellow peach decreased continuously during the shelf life, which was mainly influenced by the oxidation of polyphenols through polyphenol oxidase during fruit ripening [71]. Higher levels of TFC and TPC were maintained in yellow peach by CA at the end of the shelf life, indicating that CA significantly increased the content of TFC and TPC and delayed their loss, thus reducing the damage to the fruit by free radicals. These results are in agreement with previous reports on strawberries [72] and apples [21] as a result of the activation of PAL by CA. These results suggested that CA inhibited the oxidative decomposition of phenolic compounds, which provided yellow peach with an adequate carbon source [73] to induce defense mechanisms, and improved the quality characteristics [74]. In this sense, the previously discussed effect of CA in reducing the incidence of decay during the shelf life of yellow peach can also be interpreted as a response to the effect of CA in enhancing the capacity of antioxidant enzymes, strengthening the cell wall, and promoting the expression of plant defense genes [75].

## 5. Conclusions

This study highlights the important role of CA on the quality of yellow peach during their shelf life, and CA helps to slow down the ripening and senescence processes. The evidence presented here shows that CA can retain higher color values (L*, a*, b*, and ∆E), firmness, and inhibit the increase in WLR, DR, and BI. In addition, CA provides yellow peach with a higher clearance capacity of free radicals (DPPH, ABTS, and FRAP), and contributes to the accumulation of contents of bioactive components (TPC, TFC, neochlorogenic acid, catechin, and chlorogenic acid). Combined correlation analysis, PCA, and HCA results suggest that CA significantly improved the shelf-life quality of yellow peach, promoted its accumulation of bioactive components, improved its antioxidant capacity, and extended the shelf life. In conclusion, compared with a single low-temperature treatment, low temperature combined with CA treatment can alleviate fruit browning, improve nutrient content and antioxidant capacity, and is conducive to maintaining the shelf-life quality of yellow peach.

## Data Availability

Data are contained within the article.
